# Myocardial T1 mapping at 3 T in healthy adults: reference values and influencing factors

**DOI:** 10.1186/1532-429X-18-S1-P128

**Published:** 2016-01-27

**Authors:** Lei Zhao, Xiaohai Ma, Zhanming Fan, Tianjing Zhang

**Affiliations:** 1 grid.411606.40000000417615917Radiology, Beijing Anzhen Hospital, China, China; 2 Siemens Ltd, Beijing, China

## Background

This study aimed at analyzing the feasibility of T1 mapping at 3 T cardiovascular magnetic resonance (CMR), providing reference values and evaluating the influencing factors.

## Methods

Forty six healthy volunteers (26 females; mean age 49 ± 14 years) underwent left-ventricular T1 mapping in 3 short-axis slices at 3 T. For T1 mapping, modified Look-Locker inversion recovery sequence was used before and after injection of gadolinium contrast. T1 relaxation times were quantified for each slice and extracellular volume fraction (ECV) was calculated. The association between the T1 mapping parameters and potential influencing factors were determined using a Pearson correlation.

## Results

Mean overall left ventricle pre-contrast T1 times were 1268.0 ± 38.2 ms, post-contrast T1 times were 564.8 ± 54.8 ms, ECV were 27.3 ± 2.6%. There were no significant differences of myocardium T1 times between male and females (all p > 0.05) but ECV was slightly higher in women than men (p < 0.05). No correlation between age and T1 mapping parameters were found (all p > 0.05).

## Conclusions

Myocardial T1 reference values for the specific CMR setting are provided. T1 mapping parameters show no gender and age differences in normal individuals.

